# Persistent bradycardia requiring temporary pacing in a young adult with lyme carditis: a case report

**DOI:** 10.1186/s43044-026-00729-4

**Published:** 2026-03-09

**Authors:** Mohammad Hazique, Fnu Ekta, Sehneet Grewal, Akshat Banga, Kamran Haleem

**Affiliations:** 1 Vassar Brothers Medical Center, Poughkeepsie, USA; 2 University of Pittsburgh Medical Center, Pittsburgh, USA; 3 Mount Auburn Hospital, Cambridge, USA

**Keywords:** Lyme carditis, Tachy-Brady syndrome, Sick sinus syndrome, Transvenous pacing, Case report

## Abstract

**Background:**

Lyme disease is a common vector-borne infection that can lead to complications such as Lyme carditis (LC), particularly in untreated cases. LC can manifest as conduction abnormalities, including heart block and other arrhythmias, potentially leading to serious cardiac events.

**Case presentation:**

We report a case of a 38-year-old male with no prior medical history presenting with recurrent dizziness and bradycardia. The patient exhibited Erythema Migrans and had a history of a recent tick bite, with Lyme serology confirming the diagnosis. Despite intravenous ceftriaxone, the patient experienced persistent bradycardia and intermittent episodes of ventricular tachycardia, necessitating transvenous pacing. His condition stabilized, and he completed a 28-day antibiotic regimen, leading to full recovery.

**Conclusions:**

This case highlights the importance of recognizing Lyme carditis in endemic areas and the role of temporary pacing in managing symptomatic bradycardia. Early intervention with antibiotics and appropriate supportive measures can facilitate recovery, prevent progression, and reduce the need for permanent pacing.

## Key clinical message

Lyme carditis can present atypically, including sick sinus syndrome and tachy-brady arrhythmias, necessitating temporary pacing for symptomatic bradycardia. Early recognition, appropriate antibiotic therapy, and supportive interventions are crucial to prevent complications. Clinicians should consider Lyme carditis in endemic regions, even when atrioventricular block is absent.

## Introduction

**Fig. 1:**
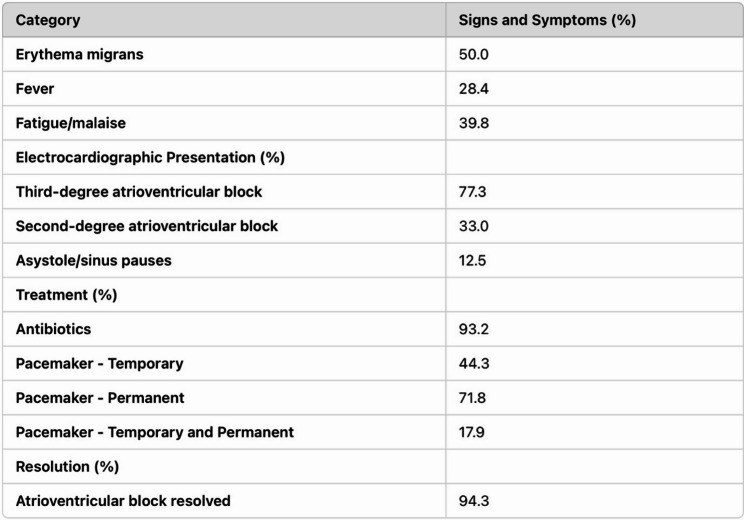
Signs and Symptoms, Electrocardiographic Presentation, Treatment, and Resolution of Patients with LC Presenting with AV nodal block

Lyme disease is the most prevalent vector-borne infection in the United States and Northern Europe, with its incidence rising significantly in recent years. In 2022, the Centers for Disease Control and Prevention (CDC) reported over 62,552 cases, nearly double the 33,000 cases in 2018. In New York State alone, 3,006 cases were diagnosed, reflecting this disease’s growing public health burden in endemic regions [1]. This rise is thought to be influenced by factors such as climate change and reforestation, which have expanded the habitats for ticks carrying Borrelia burgdorferi, the bacteria responsible for Lyme disease [2]. Acute Lyme disease typically presents with fever, generalized symptoms, and a distinctive skin lesion known as Erythema Migrans (EM) at the site of the tick bite. If untreated, the infection can disseminate to various organ systems, causing complications in the nervous system (e.g., meningitis and cranial neuritis), joints (Lyme arthritis), and the heart (Lyme carditis) [3]. Lyme carditis occurs in approximately 4–10% of untreated Lyme disease cases and arises when spirochetes infiltrate cardiac tissue, affecting all layers of the heart, including the myocardium, pericardium, and endocardium. Common cardiac manifestations include conduction abnormalities such as atrioventricular block (AVB), bundle branch block, myocarditis (Fig. 1), and pericarditis [4].

**Fig. 2:**
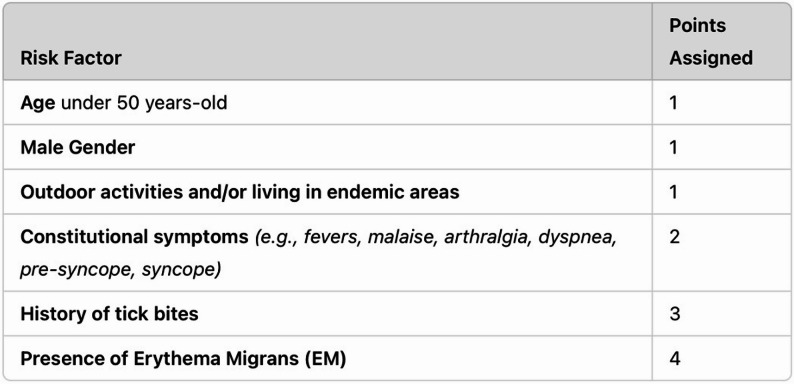
Suspicious Index in Lyme Carditis (SILC), A score of 0–2 suggests a low risk for Lyme carditis, and routine standard of care for the treatment of AVB is recommended. A score of 3–6 indicates an intermediate risk, and a score of 7–12 is considered high risk

While the early use of antibiotic therapy has significantly reduced the incidence of Lyme carditis, it remains a critical concern in untreated or late-diagnosed cases, especially in adults, while being rarely observed in children [3]. A systematic review highlighted 45 reported cases of complete heart block secondary to Lyme carditis, with 18 patients requiring temporary pacing and only two requiring permanent pacemakers [5]. The Suspicious Index in Lyme Carditis (SILC) score (Fig. 2) was developed to assist in diagnosing Lyme carditis in cases of undifferentiated high-degree AVB, offering a systematic method to evaluate risk based on clinical and epidemiological factors. However, this scoring system primarily focuses on AVB presentations [6]. This report presents a unique case of Lyme disease in a young adult male who developed sick sinus syndrome (SSS), a rare manifestation of Lyme carditis without the typical AVB presentation. Notably, the patient’s arrhythmias worsened with chronotropic agents, requiring transvenous pacing for hemodynamic support. This case highlights the importance of recognizing atypical presentations of Lyme carditis and the role of temporary pacing in managing symptomatic bradycardia. It also demonstrates the utility of the SILC score in guiding antibiotic therapy and cardiac monitoring in high-risk patients.

## Case presentation

**Fig. 3:**
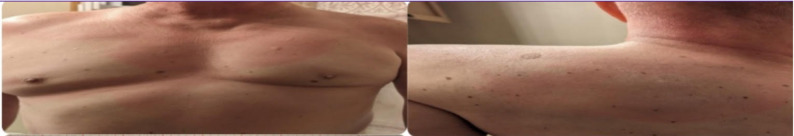
Erythema Migrans (EM) over the front and back after tick bites

**Fig. 4:**
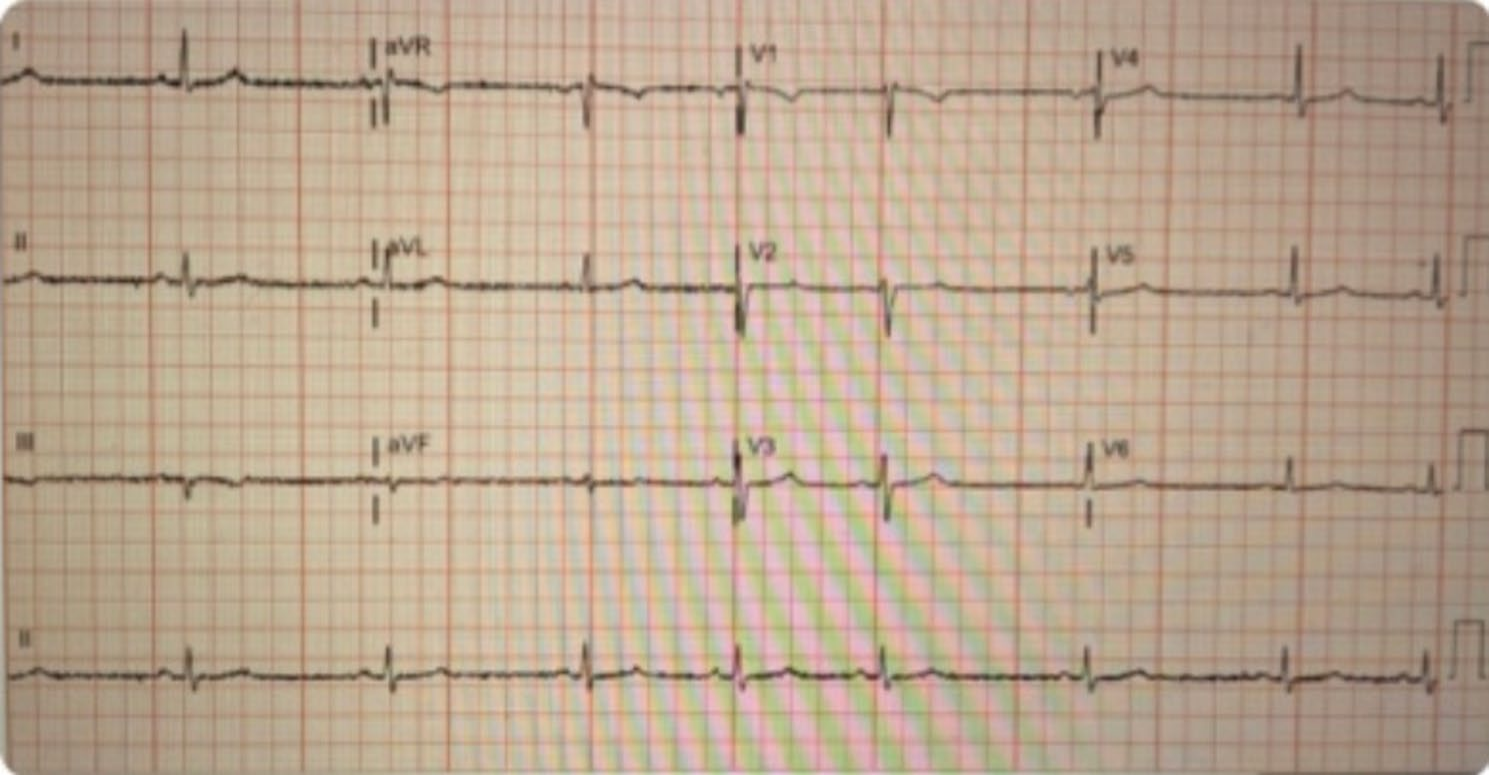
12-Lead EKG showing Sinus Bradycardia

A 38-year-old male from the Hudson Valley region of New York, a Lyme-endemic area, with no significant medical history, presented to the emergency department due to recurrent episodes of dizziness. He denied associated symptoms such as chest pain, syncope, or palpitations. On admission, his heart rate was in the 40 s, and his blood pressure measured 96/66 mmHg. Physical examination revealed erythematous rashes on his chest and back (Fig. 3). The initial ECG indicated sinus bradycardia with a heart rate of 45 bpm (Fig. 4). Aside from an elevated C-reactive protein (CRP) level of 45 mg/L, the laboratory workup was largely unremarkable. Upon further questioning, he disclosed experiencing a recent tick bite approximately 3–4 weeks prior.

### Differential diagnosis

The differential diagnoses included Lyme carditis, sick sinus syndrome, drug-induced bradycardia (e.g., beta-blockers or calcium channel blockers), hypothyroidism, myocarditis, bacterial endocarditis, and lupus or sarcoidosis. Given the patient’s geographic location, recent tick exposure, and the presence of an Erythema Migrans rash, Lyme carditis emerged as the primary considering patient elevated SILC score supported high suspicion [6].

### Investigation

**Fig. 5:**
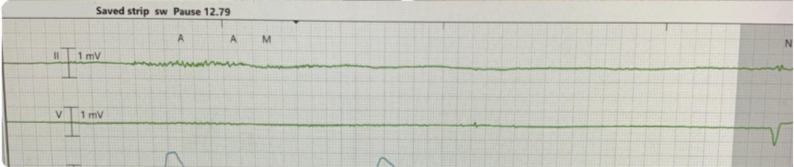
Telemetry Rhythm strip showing a significant pause around 13 s

**Fig. 6:**
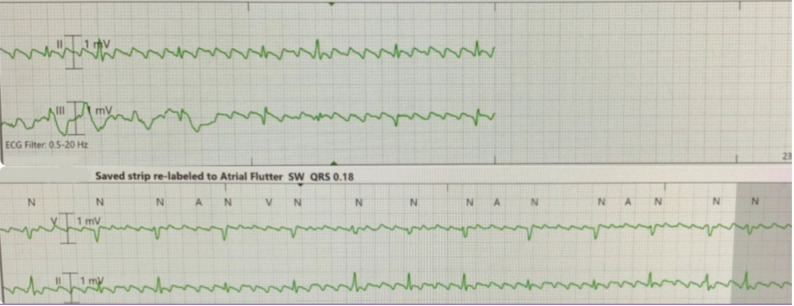
Rhythm strip showing atrial flutter on isoproterenol

Continuous telemetry monitoring revealed multiple episodes of asystole with prolonged pauses lasting up to five seconds (Fig. 5), recurrent sinus arrest and atrial flutter with variable block causing hypotension (Fig. 6). Despite these findings, the patient did not experience syncope or require cardiopulmonary resuscitation (CPR). Transthoracic echocardiogram (TTE) showed normal left ventricular systolic function, an ejection fraction of 50%, and no valvular abnormalities. Hemodynamic instability was managed with transvenous pacing to stabilize his condition. Serologic testing confirmed Lyme disease with positive IgG and IgM antibodies, verified through Western blot. Thyroid function and electrolyte levels were within normal limits, ruling out other secondary causes of bradycardia.

### Treatment

The patient was initially treated with intravenous (IV) ceftriaxone for Lyme disease. Persistent bradycardia and sinus pause necessitated the administration of isoproterenol to support the heart rate; however, this resulted in atrial flutter with variable block, this led to discontinuation of isoproterenol and initiating temporary transvenous pacing while on IV ceftriaxone, which aligns with the current ACC/AHA/HRS guidance for clinically significant cases [7], resulting in progressive clinical improvement.

### Outcome and follow-up

The transvenous pacemaker was successfully removed after ten days as the patient’s ECG normalized, with stable 1:1 conduction. He completed a 28-day antibiotic regimen, beginning with IV ceftriaxone and transitioning to oral doxycycline. At discharge, he reported no further dizziness, bradycardia, or palpitations. A four-week follow-up revealed stable sinus rhythm with a normal PR interval on ECG. Follow-up echocardiography demonstrated ongoing cardiac stability, marking the successful resolution of Lyme carditis with temporary pacing and antibiotic therapy.

## Discussion

Among individuals diagnosed with Lyme disease, cardiac manifestations in Lyme Disease (LD) were reported to affect up to 10% of cases, according to earlier research. However, more recent findings suggest a lower prevalence of Lyme Carditis with cardiac manifestations ranging from 0.3% to 4%. It is estimated that high-degree AV Block (AVB) is present in about 80% to 90% of Lyme Carditis (LC) cases [7]. There are three stages of Lyme disease [8], and patients do not necessarily exhibit symptoms stepwise or completely across these stages. Erythema Migrans (EM), often regarded as a hallmark of early localized Lyme disease, can start as a macule or papule and gradually develop into an annular lesion with central clearing, commonly referred to as a “bull’s-eye” rash [9]. Patients may present with singular or multiple lesions, which can be associated with sensations of burning, warmth, induration, pruritus, or tenderness [8]. In this case, our patient presented with large erythematous patches on the back and front of his chest, aligning with these descriptions of EM lesions (Fig. 3).

Although our patient did not exhibit constitutional flu-like symptoms prior to the development of Erythema Migrans (EM), it is common for individuals with early Lyme disease to present with symptoms such as fevers, chills, malaise, fatigue, myalgia, headache, neck stiffness, and back pain [8]. These symptoms generally last for less than a week. Lyme carditis (LC) typically develops around three weeks after EM appears, but cardiac symptoms can manifest anytime from 1 to 12 weeks after the onset of EM [8].

The persistence of Borrelia burgdorferi in the myocardial tissue exacerbates the inflammatory cascade. This process involves cytokines like tumor necrosis factor-alpha (TNF-α) and interleukin-6 (IL-6), which not only sustain the inflammatory response but also contribute to endothelial dysfunction and myocardial damage. Such disruptions in the microvascular environment of the heart are believed to play a pivotal role in the clinical manifestations of LC [10–12]. The most prevalent cardiac manifestation of LC is atrioventricular block (AVB). However, in rarer cases, patients may present with sick sinus syndrome (SSS), atrial fibrillation with rapid ventricular response, isolated tachycardia-bradycardia syndrome, myocarditis, pericarditis, pericardial effusions, endocarditis, or even cardiomegaly [4, 9]. Additionally, patients can experience reduced left ventricular ejection fraction (LVEF) [4], which is typically reversible with appropriate treatment. If timely treatment is not administered, the condition can lead to severe complications, including sudden cardiac death, though this outcome remains rare [10]. The Suspicious Index in Lyme Carditis (SILC) tool was developed with a sensitivity of 93.2% but lacks specificity data due to the absence of a control group [6]. This score aids in identifying patients with undifferentiated high-degree AVB who might be at risk for Lyme carditis, allowing for timely intervention. For patients in the intermediate to high-risk categories, serological testing is recommended for Lyme disease, empiric antimicrobials, possible pacemaker insertion is recommended for patients with symptomatic bradycardia, and admission for close monitoring [13–15].

Our patient exhibited no signs of AV block (AVB) but had a SILC Risk Score of 10, which accounted for his age, male gender, outdoor exposure, a history of tick bites, and the presence of Erythema Migrans (EM), indicating a high risk for Lyme carditis. However, it is essential to note that the SILC Risk Score was explicitly designed to assess risk in patients with AVB [6]. Therefore, further studies are necessary to determine whether this tool is equally effective for stratifying risk in patients who present with bradycardia but do not have AV block.

A Two-step serological testing process for diagnosing Lyme disease. The first step involves an enzyme immunoassay (EIA) or indirect immunofluorescence assay (IFA). If these tests are negative, further testing is not typically required unless clinical suspicion of Lyme carditis remains high, in which case empiric antibiotic treatment may be considered. If the initial tests are positive or equivocal, further testing depends on the symptom duration. For symptoms present for 30 days or less, IgG and IgM Western blot tests for B. burgdorferi antigens are recommended. For symptoms persisting beyond 30 days, only an IgG Western blot should be performed to detect antibodies [16]. Endomyocardial biopsy remains the gold standard for diagnosing myocarditis and is particularly crucial in complex cases [17]. For bradyarrhythmia with hemodynamic compromise, temporary transvenous pacing is appropriate per ACC/AHA/HRS bradycardia guidelines [6], with permanent pacing deferred when a reversible cause is likely. These principles extend to significant sinus arrest/SND when symptomatic or unstable, as in our case temporary pacing helped with recovery [18]. Implementing temporary permanent pacing (TPP) can accelerate recovery and facilitate earlier patient discharge. Although TPP has been used in only a limited number of Lyme carditis cases, it is recommended for managing symptomatic high-degree AV block and Sick Sinus Syndrome in early disseminated Lyme disease [18].

The primary treatment options for early localized Lyme disease are doxycycline, amoxicillin, or cefuroxime. In children under eight years old, pregnant women, and lactating mothers, doxycycline should be avoided due to the risk of teeth discoloration, with amoxicillin or cefuroxime being preferred [3]. For early disseminated Lyme disease with complications like atrioventricular block (AVB), myopericarditis, meningitis, or radiculopathy, ceftriaxone is recommended as first-line therapy, with doxycycline as an oral alternative [3]. For patients experiencing symptomatic bradycardia or second/third-degree AVB, a temporary pacemaker can be utilized alongside antibiotics [13–15]. For late disseminated Lyme disease without cardiac involvement, doxycycline, amoxicillin, or cefuroxime remain first-line treatments [3]. Symptom relief can be achieved with nonsteroidal anti-inflammatory drugs (NSAIDs), disease-modifying antirheumatic drugs (DMARDs), or intra-articular steroid injections [19].

## Conclusion

We suggest that transvenous pacing is a practical and effective approach for managing symptomatic bradycardia or sick sinus syndrome (SSS) in Lyme carditis when there is a lack of response to antibiotic therapy and an increased risk of cardiac arrest. This method avoids unnecessary permanent pacemaker implantation in conjunction with the appropriate antimicrobial treatment. Compared to conventional temporary pacemakers, transvenous pacing allows for earlier mobilization, enhancing patient recovery and comfort.

## Data Availability

No datasets were generated or analysed during the current study.

## Abbreviations

ECG: Electrocardiogram
CRP: C-reactive protein
IV: Intravenous
TTE: Transthoracic echocardiogram
SILC: Suspicious index in lyme carditis
EM: Erythema migrans
SSS: Sick sinus syndrome
AVB: Atrioventricular block
SIRS: Systemic inflammatory response syndrome
LVEF: Left ventricular ejection fraction
NSAIDs: Nonsteroidal anti-inflammatory drugs
DMARDs: Disease-modifying antirheumatic drugs
TPP: Temporary permanent pacemaker
SILC: Suspicious index in lyme carditis
IV: Intravenous
